# Ezrin and Moesin Are Required for Efficient T Cell Adhesion and Homing to Lymphoid Organs

**DOI:** 10.1371/journal.pone.0052368

**Published:** 2013-02-28

**Authors:** Emily J. H. Chen, Meredith H. Shaffer, Edward K. Williamson, Yanping Huang, Janis K. Burkhardt

**Affiliations:** Department of Pathology and Laboratory Medicine, The Children's Hospital of Philadelphia and the Perelman School of Medicine at the University of Pennsylvania, Philadelphia, Pennsylvania, United States of America; University of California, San Francisco, United States of America

## Abstract

T cell trafficking between the blood and lymphoid organs is a complex, multistep process that requires several highly dynamic and coordinated changes in cyto-architecture. Members of the ezrin, radixin and moesin (ERM) family of actin-binding proteins have been implicated in several aspects of this process, but studies have yielded conflicting results. Using mice with a conditional deletion of ezrin in CD4+ cells and moesin-specific siRNA, we generated T cells lacking ERM proteins, and investigated the effect on specific events required for T cell trafficking. ERM-deficient T cells migrated normally in multiple *in vitro* and *in vivo* assays, and could undergo efficient diapedesis *in vitro*. However, these cells were impaired in their ability to adhere to the β1 integrin ligand fibronectin, and to polarize appropriately in response to fibronectin and VCAM-1 binding. This defect was specific for β1 integrins, as adhesion and polarization in response to ICAM-1 were normal. *In vivo*, ERM-deficient T cells showed defects in homing to lymphoid organs. Taken together, these results show that ERM proteins are largely dispensable for T cell chemotaxis, but are important for β1 integrin function and homing to lymphoid organs.

## Introduction

Adaptive immunity is highly dependent on lymphocyte adhesion and migration. In the absence of infection, lymphocytes continually patrol the body, circulating between the blood and secondary lymphoid organs. Preferential homing to secondary lymphoid organs is directed by specific chemokines such as CCL19 and CCL21 [1], [2], [3]. In response to infection or other inflammatory stimuli, activated effector lymphocytes migrate to sites of inflammation [4]. This process is directed by an array of chemokines, which vary depending on nature and location of the inflammatory signals [5], [6], [7]. Defects in T cell adhesion and migration, for example in patients with Leukocyte Adhesion Deficiencies, result in severe immunodeficiency [8]. Conversely, proteins involved in lymphocyte adhesion and migration are important targets for immunotherapies in cancer, autoimmunity, and transplantation settings [9].

Lymphocyte recirculation and homing to lymphoid organs involve a highly programmed sequence of events to permit passage across endothelial barriers at the appropriate sites. Within the bloodstream, T cells possess well-formed microvilli enriched at their tips in low-affinity adhesion molecules such as L-selectin [10]. As T cells pass through specialized high endothelial venules (HEVs) within lymph nodes, these selectins interact with ligands on endothelial cells, inducing tethering and rolling of lymphocytes along the vascular wall [11]. In response to chemokines expressed on the endothelial cells, lymphocyte microvilli collapse, promoting the binding of the integrins LFA-1 and VLA-4 to their ligands ICAM-1 and VCAM-1, respectively [12], [13]. In parallel, outside-in signaling events upregulate integrin affinity and avidity, leading to lymphocyte arrest and firm adhesion on the endothelium [14], [15], [16]. Once bound, T cells migrate along the endothelium in search of an ideal location for transmigration [17]. Most often, T cells pass between endothelial cells, a process that also depends on integrin engagement [18], [19], [20], [21]. Once in the lymph node, T cells migrate through the dense cellular network in search of cognate antigen presented by antigen presenting cells.

This multistage process requires dynamic and highly organized actin cytoskeletal changes. In addition to organizing the microvilli that mediate tethering and rolling [22], the actin cytoskeleton is important for generation of protrusive structures that allow passage through endothelial barriers [23]. Moreover, the actin cytoskeleton plays a fundamental role in organizing cell polarity. T cells migrating on the endothelial wall and within lymphoid tissues have a clear leading edge and trailing uropod, and this polarized morphology is thought to maintain persistent movement in response to chemotactic stimuli [24], [25]. One set of proteins that play a central role in T cell trafficking is the Ezrin, Radixin and Moesin (ERM) family of actin-binding proteins. ERM proteins organize microvilli and localize to the uropods of migrating cells [26], [27], [28], and they promote cell rigidity by linking plasma membrane proteins to the underlying actin cytoskeleton [29], [30]. ERM proteins bind to cell surface and cytoplasmic molecules via an N-terminal FERM domain, and tether these molecules to the actin cytoskeleton via a C-terminal actin-binding domain [31], [32]. This linker activity is regulated by intramolecular interactions between the FERM and actin-binding domains, which prevent binding to cargo proteins and actin [33], [34]. Autoinhibition is relieved by interaction with PIP2 in the plasma membrane and by phosphorylation of a conserved threonine in the actin-binding domain [35], [36]. Physiological function of ERM proteins is highly dependent on cycling between the active and auto-inhibited conformations. At steady state, approximately 50% of the ERM proteins in a T cell are in an active, phosphorylated form [37]. Chemokine stimulation induces transient ERM protein inactivation by PIP2 hydrolysis and dephosphorylation at the regulatory threonine [12], [38]. This process leads to microvillar collapse and permits reorganization of ERM binding proteins and their binding partners at the cell surface [12]. Since active ERM proteins confer rigidity to the cell cortex, transient inactivation also permits cell deformation early in a chemotactic response. Rephosphorylation, carried out in part by lymphocyte-oriented kinase (LOK) [39], occurs within 10 minutes of chemokine exposure [12], restoring rigidity and stabilizing the new cortical architecture.

Several studies demonstrate that ERM proteins are important for lymphocyte adhesion and migration. However, the literature is divided as to whether these proteins promote or impede specific aspects of this process [30], [39], [40]. Until now, analysis of ERM protein function has relied on the use of constitutively active phospho-mimetic mutants, or mutations in kinases that alter ERM protein activation status. While this is a valuable approach, interpretation of such studies can be problematic because constitutively active mutants (or overexpression of LOK) effectively lock the cell cortex in a crosslinked state and can saturate binding partners. This can yield complex phenotypes that may not reflect the normal function of cycling ERM proteins. Hypo-phosphorylating ERM proteins by deleting LOK circumvents this problem, but carries the risk that other kinase targets will also be affected. The effects of abolishing expression of ERM proteins on T cell adhesion and migration have not been directly tested, in part because T cells express both ezrin and moesin, and these highly homologous proteins show overlapping function [37]. In this study, we used a combination of gene deletion and siRNA approaches to generate T cells deficient for both ezrin and moesin. We find that these cells show defects in integrin-dependent adhesion and in homing to lymphoid organs.

## Results

Studies utilizing constitutively active ezrin mutants or other conditions that promote hyper-activation of ERM proteins consistently show effects on lymphocyte chemotaxis, but specific results are conflicting. Some studies show that ERM activation inhibits chemotaxis [30], [41], while others demonstrate enhancement [39], [40]. Since cycling of ERM proteins is central to their function, such studies can be difficult to interpret. We therefore re-evaluated the role of ERM proteins in T cell migration using T cells lacking these proteins. To achieve this, we used T cells from ezrin^flox/flox^ mice expressing Cre under the CD4 promoter and suppressed moesin expression with siRNA (SiM). ERM-deficient T cells prepared using this approach have been described previously [37]; these cells lack ezrin expression, and express moesin at ∼10–20% of endogenous levels. For brevity, these cells are referred to as Ez^−/−^Mo^SiM^, and control cells generated from ezrin^flox/flox^ mice and transfected with control siRNA are termed Ez^+/+^Mo^SiC^.

### ERM-deficient T cells chemotax in response to CCR7 ligands

**Figure 1:**
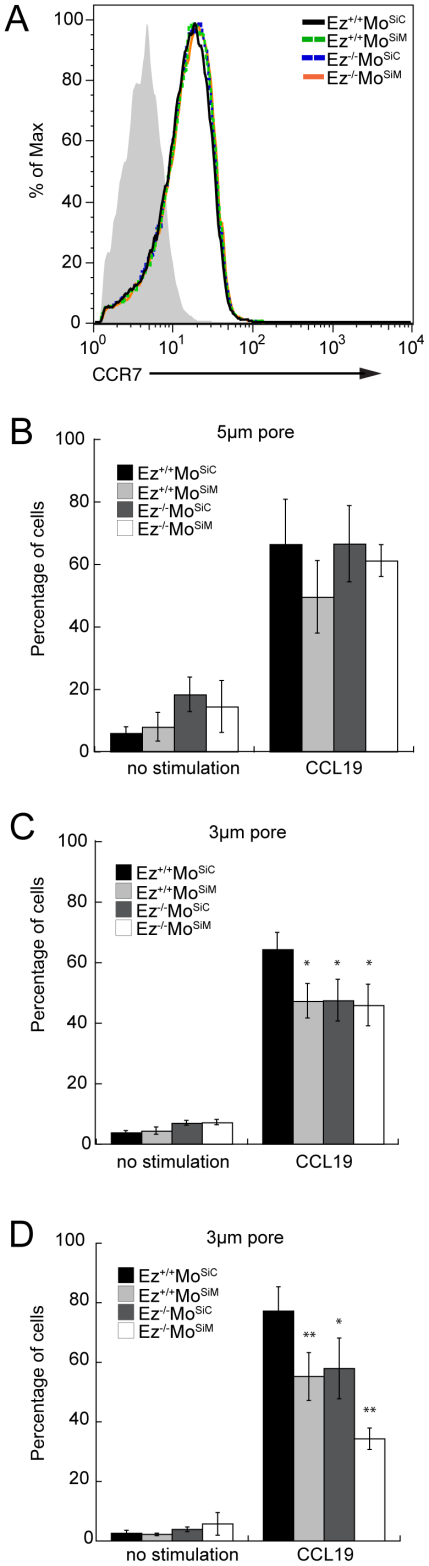
ERM-deficient T cells can chemotax efficiently *in vitro*. Wild-type T cells expressing both ezrin and moesin (Ez^+/+^Mo^SiC^), T cells lacking moesin (Ez^+/+^Mo^SiM^), T cells lacking ezrin (Ez^−/−^Mo^SiC^), or T cells lacking both ezrin and moesin (Ez^−/−^Mo^SiM^), were prepared as described in Materials and Methods. (A) Cells were stained for CCR7 and assessed by flow cytometry. Filled histogram, isotype control. (B–D) Wild-type or the indicated ERM-deficient T cells were placed in a transwell assay in the absence or presence of 40 nM CCL19 for 2 hours. Cells that migrated across a 5 µm (B) or 3 µm (C and D) pore membrane to the bottom well were quantified, and are presented as the percentage of input. Data are mean ± StDev of quadruplicate wells from one experiment, representative of three experiments. *p<0.05, **p<0.005.

As shown in Figure 1A, wild-type and ERM-deficient T cells expressed comparable surface levels of the chemokine receptor CCR7. We therefore asked if ERM protein deficiency affects chemotactic responses to CCR7 ligands using an *in vitro* transwell assay. In addition to testing cells deficient for both ezrin and moesin, cells expressing only ezrin or only moesin were also tested to address the possibility of redundancy. As shown in Figure 1B, in the absence of chemokine, a low percentage of wild-type, single deficient and double deficient T cells were able to cross a 5 µm pore membrane. In the presence of chemokine in the lower chamber, efficient chemotaxis was observed for all cell populations. This demonstrates that ERM-deficient T cells are able to respond to CCR7 ligands, and can chemotax efficiently. Since ERM proteins have been implicated in regulating T cell cortical rigidity and tension [29], [30], we reasoned that ERM-deficient T cells might show enhanced ability to cross a constricted barrier. To test this, we repeated the assay using transwell chambers with 3 µm pores. Surprisingly, T cells lacking ERM proteins migrated less efficiently than wild-type cells through the smaller pores (Figure 1C). In some experiments, T cells lacking both ezrin and moesin showed a more profound phenotype than T cells lacking only one ERM protein (Figure 1D), consistent with the idea that these proteins have partially overlapping functions in T cells [37].

**Figure 2:**
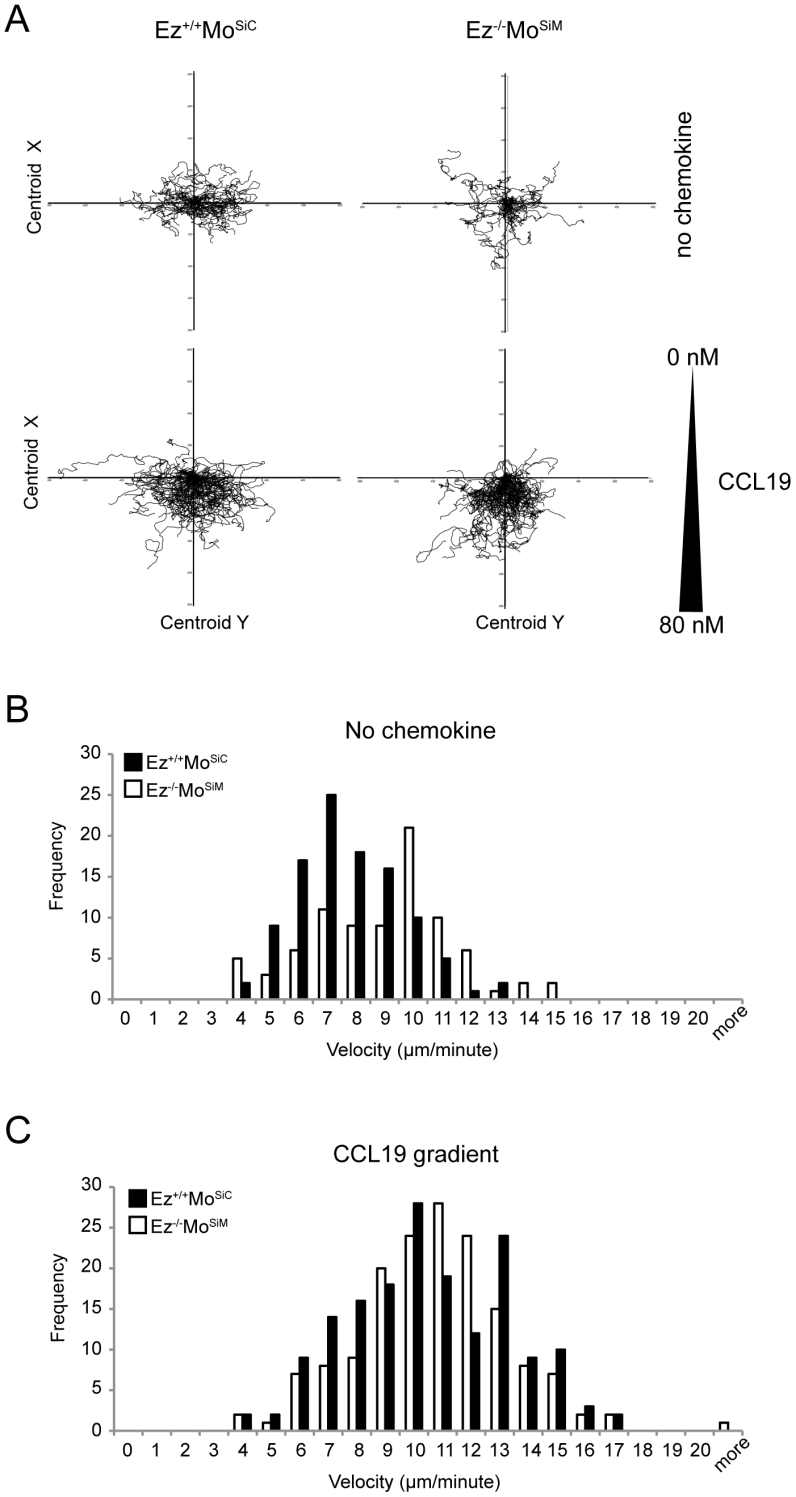
ERM-deficient T cells can migrate efficiently in confined spaces. A) Wild-type or ERM-deficient T cells were placed in a 5 µm pore collagen gel in the absence (top panels) or presence (bottom panels) of a CCL19 gradient, and cell migration was imaged for 4 hours at 37°C. Tracks of individual cells are presented with the same point of origin. Data are representative of three experiments. Quantitative analysis is presented in Tables 1 and 2. B and C) Frequency of individual cell velocities in (A) in the absence (B) or presence (C) of chemokine. Data are representative of at least three collagen gels per condition in two independent experiments.

To further assess the ability of ERM-deficient T cells to migrate within confined spaces, we tested chemotaxis in a three-dimensional (3D) setting. In collagen gels with an average pore size of 5 µm and in the absence of chemokine, ERM-deficient T cells migrated as efficiently as wild-type T cells (Figure 2A, top panels). Although no differences between the two populations were observed in track length, meandering index, or directionality, the average velocity of ERM-deficient T cells was modestly increased in the absence of chemokine (Table 1). Plotting the data as a frequency histogram (Figure 2B) revealed that the velocities of randomly migrating ERM-deficient T cells are highly variable, and the population lacks the Gaussian distribution observed in wild-type cells. Wild-type and ERM-deficient T cells were also tested for chemotaxis in 3D collagen gels in response to a gradient of CCL19. As anticipated, exposure of wild-type T cells to chemokine triggered an increase in velocity and enhanced directional migration (Figure 2A, bottom panels, and Table 2). Consistent with our findings using the transwell assay, ERM-deficient T cells also chemotaxed efficiently. As shown in Table 2, chemotaxing ERM-deficient T cells exhibited velocities, meandering indices and track lengths similar to wild-type cells. Frequency histograms showed that ERM-deficient and wild-type T cells exhibited comparable Gaussian distributions (Figure 2C). Similar results were obtained if we increased the collagen concentration to yield gels with a predicted average pore size of 2 µm (data not shown). Taken together, these findings show that loss of ERM protein expression does not impair T cell migration or chemotaxis *per se*, though it does affect movement through confined spaces in some experimental settings.

**Table 1:** Random migration in collagen gels.

	Ez^+/+^Mo^SiC^ (N = 105)		Ez^−/−^Mo^SiM^ (N = 85)		
No chemokine	Mean	± Stdev	Mean	± Stdev	T-test
Track length (µm)	1024.02	473.33	1159.06	639.36	n.s
Velocity (µm/min)	7.21	1.83	8.53	2.46	**p<0.005**
Meandering index^a^	0.29	0.14	0.28	0.15	n.s
Angle^b^	95.65	43.63	99.13	46.99	n.s.
Bearing^c^	172.26	94.65	173.63	93.32	n.s.

a *Meandering index* is defined as net displacement divided by track length, such that migration in a straight line gives a value of 1, while greater meandering leads to values closer to zero.

b *Angle* measures migration along the Y-axis. Values range from 0° to 180°, such that random migration should give a value of 90°.

c Bearing measures migration along the X-axis. Values range from 90°–270°, such that random migration should give a value of 180°.

**Table 2:** Chemotaxis in collagen gels.

	Ez^+/+^Mo^SiC^ (N = 168)		Ez^−/−^Mo^SiM^ (N = 158)		
80 nM CCL19	Mean	± Stdev	Mean	± Stdev	T-test
Track length (µm)	957.64	429.37	976.23	488.90	n.s.
Velocity (µm/min)	9.99	2.76	10.25	2.67	n.s.
Meandering index^a^	0.35	0.14	0.36	0.15	n.s.
Angle^b^	134.16	35.35	145.16	27.35	**p<0.005**
Bearing^c^	174.85	57.66	179.00	44.28	n.s.

a *Meandering index* is defined as net displacement divided by track length, such that migration in a straight line gives a value of 1, while greater meandering leads to values closer to zero.

b *Angle* measures migration along the Y-axis (parallel to the chemokine gradient). Values range from 0° to 180°, such that direct migration toward the chemokine gives a value of 180°.

c Bearing measures migration along the X-axis (perpendicular to the chemokine gradient). Values range from 90°–270°, such that perfect migration toward the chemokine (no side-to-side wandering), gives a value of 180°.

### ERM proteins are required for efficient responses to β1, but not β2, integrin ligands

**Figure 3:**
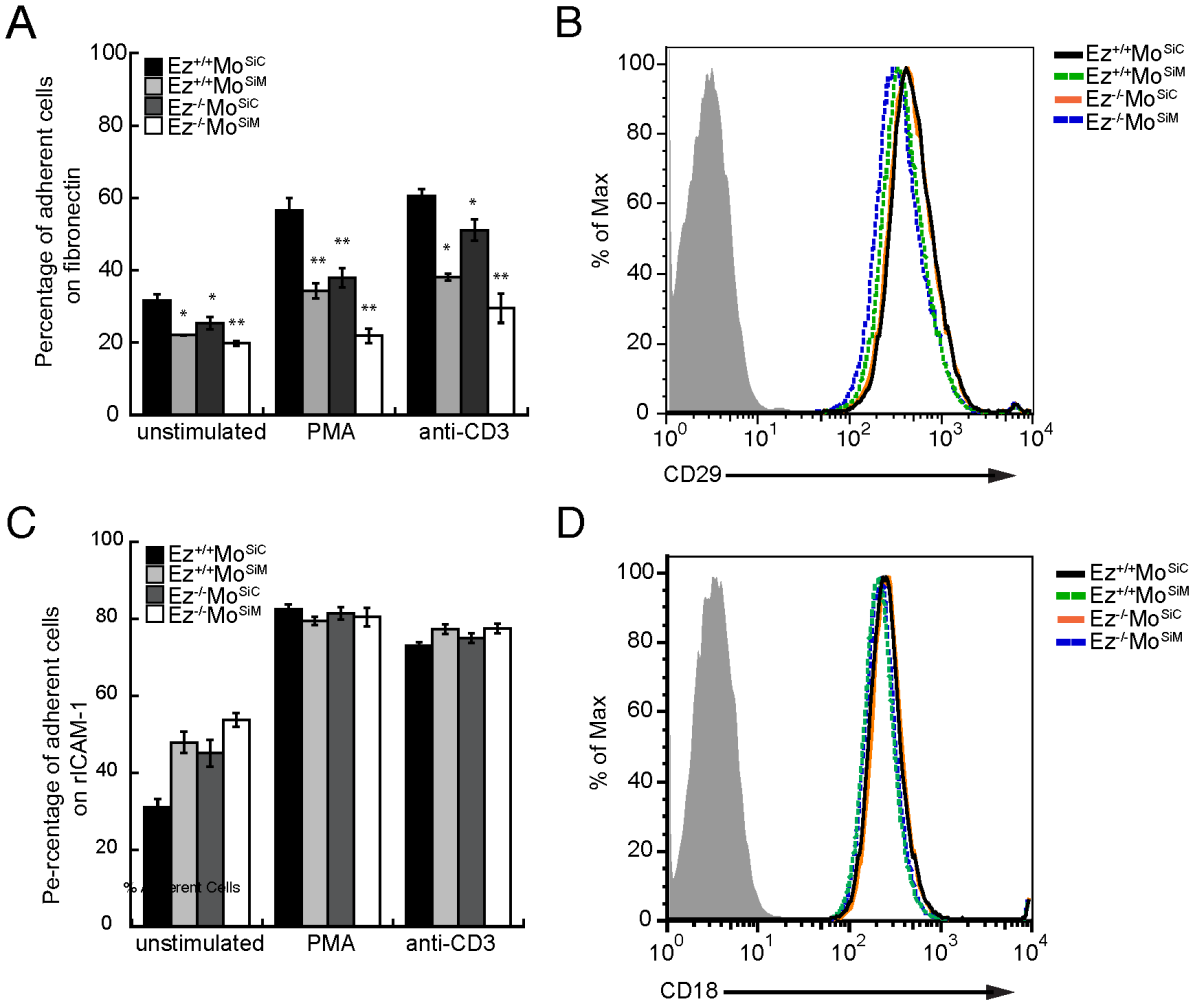
ERM proteins are required for efficient adhesion to β1, but not β2 integrin ligands. (A and C) Wild-type or the indicated ERM-deficient T cells were stained with Calcein-AM and settled in 96-well plates coated with fibronectin (A) or rICAM-1 Fc (C). Cells were either left unstimulated or stimulated as indicated, non-adherent cells were washed off, and fluorescence was measured using a microplate reader. Adherent cells are shown as a percentage of input. Data shown are means ± StDev from triplicate wells in one experiment, representative of three individual experiments. * p<0.05, **p<0.005. (B and D) Cells were stained with either anti-CD29 (B) or anti-CD18 (D) and analyzed by flow cytometry to assess surface integrin levels.

Overexpression of constitutively active ERM proteins has been shown to enhance adhesion to integrin ligands [30], [40]. To ask if deficiency in ERM proteins enhances or inhibits adhesion, wild-type, single deficient and double deficient T cells were allowed to settle on surfaces coated with the β1 integrin ligand fibronectin, non-adherent cells were washed away and specific binding was assessed. As anticipated, wild-type T cell blasts showed basal adhesion in the absence of stimulation, and adhesion was enhanced by treatment with either PMA or anti-CD3 (Figure 3A). ERM-deficient T cells showed significantly lower basal binding to fibronectin than wild-type cells. Moreover, activation-dependent binding of ERM-deficient T cells was strikingly lower than that of wild-type cells. Intermediate, but still statistically significant, defects were observed in T cells lacking only ezrin or moesin, pointing to overlapping function of these proteins in promoting β1 integrin-dependent adhesion. The diminished adhesion we observed in ERM-deficient T cells was not due to changes in β1 integrin expression; all cell populations expressed comparable surface levels of the β1 chain CD29 (Figure 3B). Somewhat surprisingly, parallel studies using the β2 integrin ligand ICAM-1 did not reveal ERM-protein dependent binding. As shown in Figure 3C, both basal and activation-induced binding of ERM-deficient T cells to ICAM-1 were comparable to that of wild-type T cells. As expected, ERM-deficient T cells expressed normal levels of the β2 chain CD18 (Figure 3D).

**Figure 4:**
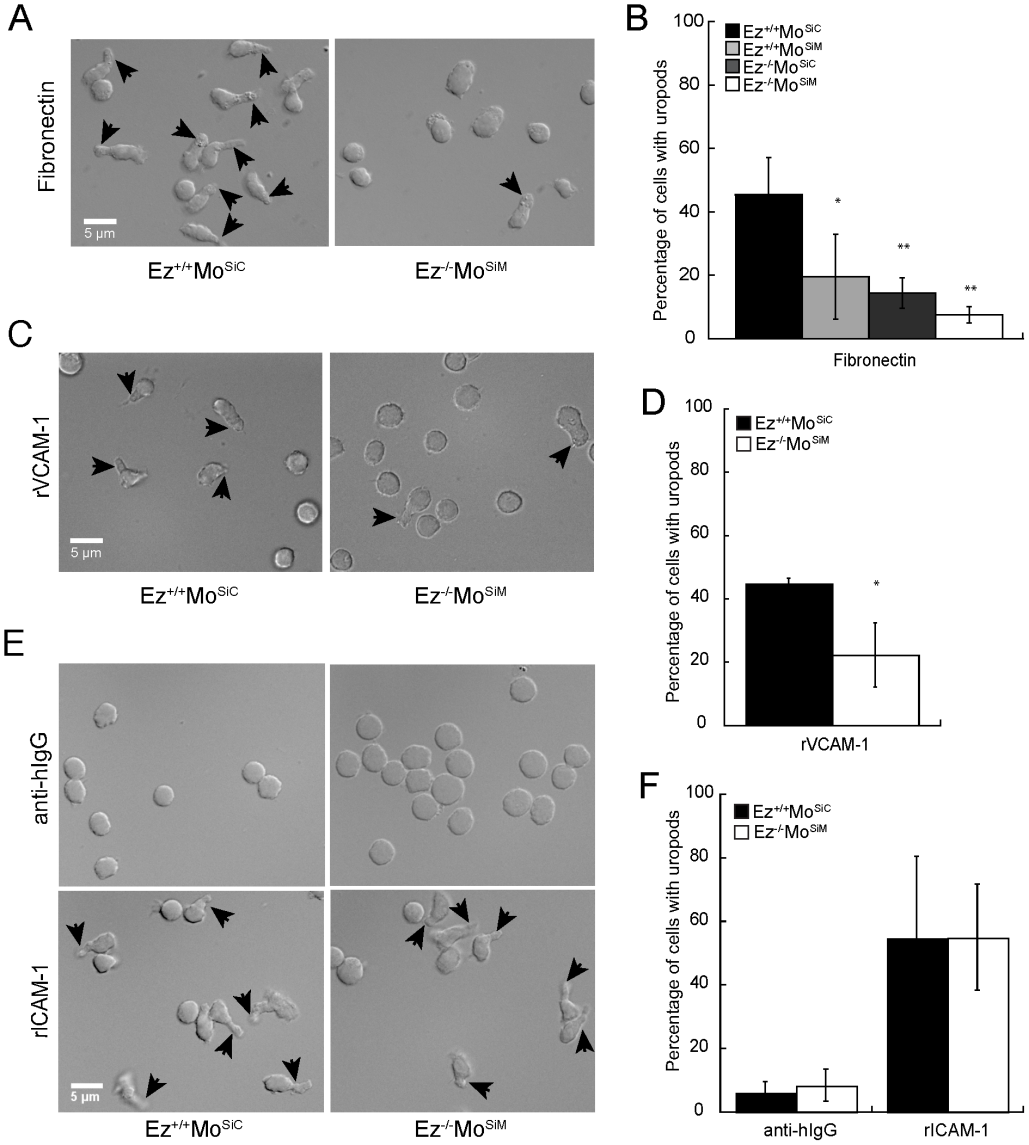
ERM proteins are required for uropod formation in response to β1, but not β2 integrin engagement. (A) Wild-type or ERM-deficient T cells were allowed to interact with fibronectin-coated glass coverslips, fixed and imaged using DIC optics. Uropods are marked with arrows. (B) Quantitation of uropod formation in cells analyzed in (A). (C) Wild-type or ERM-deficient T cells were allowed to interact with glass coverslips coated with anti-human IgG followed by rVCAM-1 Fc and imaged as in (A). (D) Quantitation of uropod formation in cells analyzed in (C). (E) Wild-type or ERM-deficient T cells were allowed to interact with glass coverslips coated with anti-human IgG alone (top panels) or together with rICAM-1 Fc (bottom panels), and imaged as in A. (F) Quantitation of uropod formation in cells analyzed in (E). Data in B, D and F represent mean ± StDev of at least 5 coverslips, with 50–100 cells each, from 2–7 independent experiments. *p<0.05, **p<0.005.

Outside-in signals from engaged integrins can induce T cell polarization to form a leading edge and trailing uropod, setting the stage for migration [42], [43]. ERM proteins are localized to the T cell uropod and have been implicated in maintenance of T cell polarity [24], [30], [40], [44]. We therefore asked if ERM-deficient T cells polarize appropriately in response to fibronectin binding. T cells were settled on fibronectin-coated glass coverslips, and uropod formation was assessed by DIC microscopy. As anticipated, wild-type T cells formed a characteristic “hand mirror” shape, with a rounded cell body and a constricted trailing uropod (Figure 4A, uropods indicated with arrows). In contrast, ERM-deficient T cells were rounded or elongated, but rarely exhibited a clear uropod. Quantitative analysis showed that uropod formation was significantly reduced in T cells lacking ezrin or moesin alone, and profoundly reduced in cells lacking both ERM proteins (Figure 4B). This defect was not specific to fibronectin, since inefficient uropod formation in ERM-deficient T cells was also observed in response to VCAM-1, another β1 integrin ligand (Figure 4C and D). Since β2-integrin mediated binding to ICAM-1 was intact, we asked whether ERM-deficient T cells form uropods efficiently in response to ICAM-1. T cells were settled on coverslips coated with anti-hIgG and ICAM-1 Fc or with anti-hIgG alone. In the absence of integrin ligand, wild-type and ERM-deficient T cells did not form uropods (Figure 4E and F). However, in the response to ICAM-1, both wild-type and ERM-deficient T cells formed uropods at comparable frequencies. Taken together, these studies show that ERM proteins are specifically required for adhesion and uropod formation in response to β1 integrin ligands. Though ERM proteins have been implicated as structural uropod components, our data showing that ERM-deficient T cells can generate a normal uropod in response to ICAM-1 suggest that the defects in uropod formation reflect abnormal outside-in signaling through β1 integrins rather than an inability to form a uropod *per se*. Consistent with this, we have observed that proliferating ERM-deficient T cells also readily form uropods in tissue culture (data not shown).

### ERM proteins are required for T cell homing to peripheral lymphoid organs

**Figure 5:**
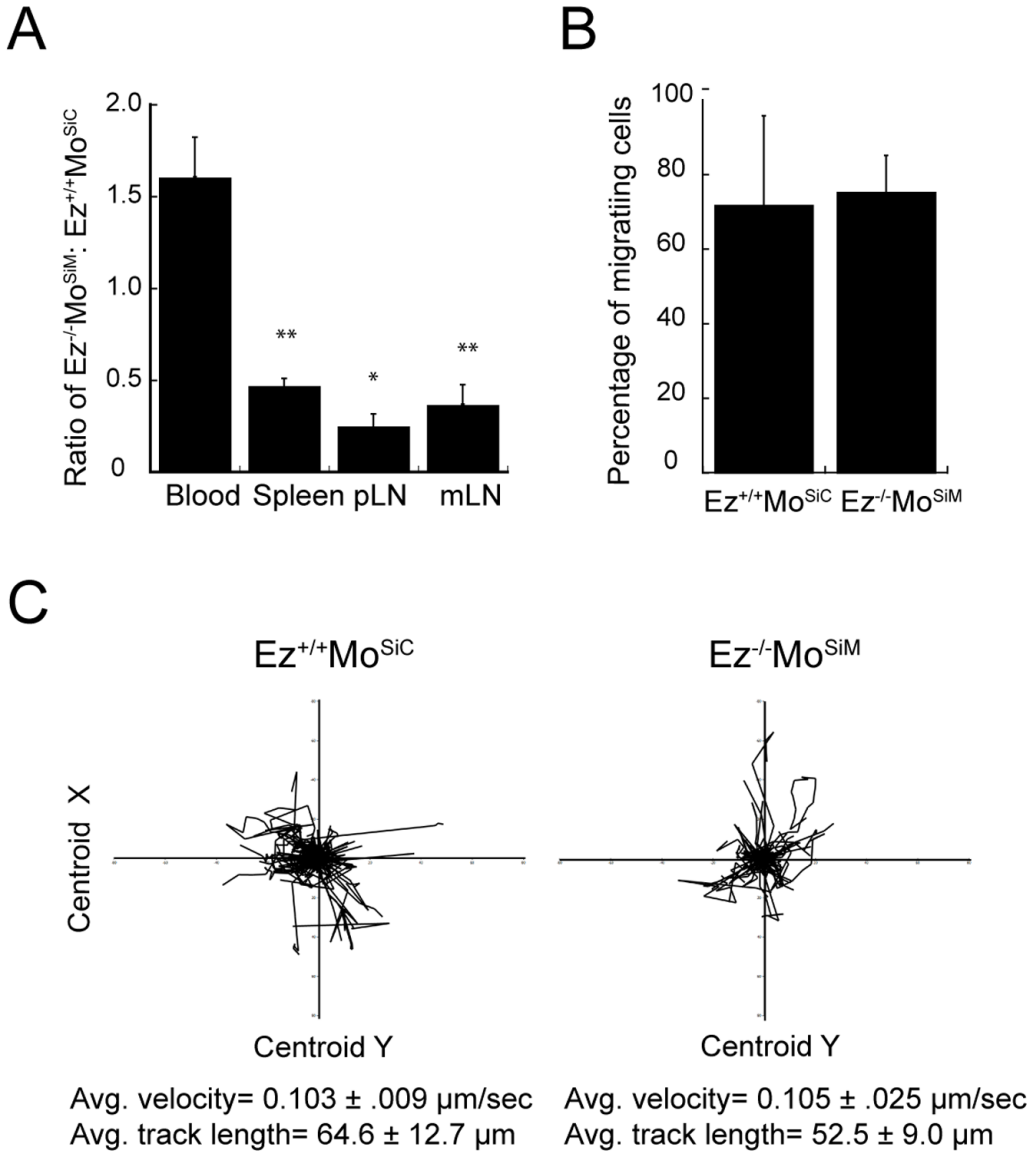
ERM proteins are required for T cell homing to peripheral lymphoid organs. Wild-type and ERM-deficient T cells were differentially labeled with CFSE and CMTMR, mixed in a 1∶1 ratio and injected into the tail veins of C57Bl/6 hosts. (A) Blood, spleen, and peripheral and mesenteric lymph nodes were collected 1 hour after injection, and cell suspensions were analyzed by flow cytometry. The ratio of adoptively transferred ERM-deficient to wild type T cells is shown. Data represent mean ± StDev from 5 mice in one experiment, representative of four experiments. * p<0.05, **p<0.005. (B and C) Lymph nodes were harvested 1 hour after injection for multi-photon imaging, and cell migration was tracked. (B) The percentage of cells showing active migration, defined as detailed in Materials and Methods. (C) Tracks, average velocities and average track lengths from one representative lymph node. Data represent mean ± StDev of three experiments.

T cells trafficking *in vivo* involves the coordination of adhesion and chemotaxis, as well as the ability to squeeze through tissue barriers. To assess the ability of ERM-deficient T cells to migrate to secondary lymphoid organs *in vivo*, we used a competitive homing assay. Wild-type and ERM-deficient T cells were each fluorescently labeled, mixed in a 1∶1 ratio, and co-injected intravenously into wild-type recipient mice. After 1 hour, recipient mice were sacrificed, and blood, peripheral lymph nodes and spleens were harvested and analyzed by flow cytometry. As shown in Figure 5A, significantly reduced numbers of ERM-deficient cells were found in peripheral lymph nodes and spleen compared to wild-type cells. Conversely, the proportion of ERM-deficient cells remaining in the blood was significantly higher. These data show that expression of ERM proteins is required for efficient homing of T cells to secondary lymphoid organs.

Although homing of ERM-deficient T cells was inefficient, some cells did enter secondary lymphoid organs. To ask whether these cells show migratory defects, we visualized the behavior of wild-type and ERM-deficient cells within the lymph node stroma using multi-photon microscopy of *ex vivo* lymph nodes (Video S1). We found that the majority of both wild-type and ERM-deficient T cells actively migrated within the lymph node (Figure 5B). No differences were observed in directionality, track length, or velocity (Figure 5C). This result is consistent with our findings in the collagen gel assay, showing that ERM proteins are not required for migration in a three-dimensional setting. We note, however, that this analysis could only be performed on the minority of ERM-deficient T cells that reach the lymph node. As discussed further below, these cells may represent a population that expresses significant levels of residual moesin. In any case, these studies clearly show that ERM proteins are required for efficient T cell homing *in vivo*.

### ERM-deficient T cells undergo diapedesis efficiently

**Figure 6:**
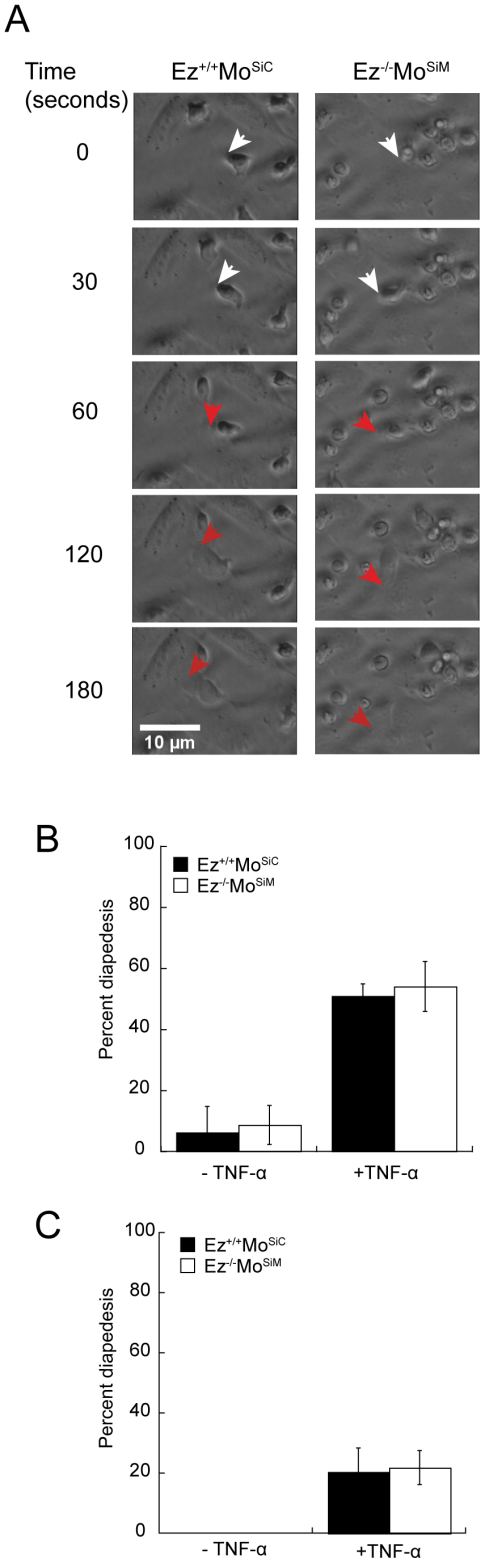
ERM-deficient cells undergo efficient diapedesis *in vitro*. (A,B) Diapedesis under static conditions. Confluent monolayers of 3B-11 endothelial cells were pre-treated with or without TNFα to upregulate VCAM-1, and wild-type or ERM-deficient T cells were added to the apical surfaces. Cells were imaged in an environmental chamber every 30 seconds for 2 hours. (A) DIC images of cells undergoing diapedesis. Arrowheads indicate leading edges of migrating T cells. White arrows indicate cells migrating along the apical surface of endothelial cells; the same cells are marked with red arrows after passing below the endothelial cell layer. (B) Quantitative analysis of assays carried out as in A. Cells undergoing diapedesis are quantified as a percentage of total moving cells. Data represent mean ± StDev from six experiments. (C) Diapedesis under shear flow conditions. Confluent monolayers of 3B-11 endothelial cells were grown in flow chambers, pre-treated with or without TNFα to upregulate VCAM-1, and wild-type or ERM-deficient T cells were added to the apical surfaces and allowed to interact with endothelial cells under shear stress of 0.5 dyne/cm^2^. Cells were imaged every 30 seconds for 1 hour, and the percentage of moving cells that underwent diapedesis was determined. Data represent mean ± StDev from three experiments.

In order to enter lymph nodes, T cells must first undergo integrin-mediated adhesion to endothelial cells, and then extravasate by passing through the constricted spaces between endothelial cells, a process termed diapedesis. We therefore assessed the ability of ERM-deficient T cells to undergo diapedesis (Figure 6A). Since ERM-deficient T cells were impaired in their ability to respond to β1 integrin ligands, we selected an endothelial cell line that expresses the β1 integrin ligand VCAM-1 in response to inflammatory stimuli, but lacks β2 integrin ligands ([45] and data not shown). Confluent monolayers of murine 3B-11 endothelial cells were either left untreated or treated with TNF-α to induce upregulation of VCAM-1 (confirmed by flow cytometry, data not shown). Control or ERM-deficient T cells were then placed on top of each monolayer, and diapesesis was imaged using DIC microscopy. As shown in Figure 6A and Video S2, T cells migrating on top of the endothelial monolayer are highly refractile, while cells that have passed beneath the monolayer are readily identifiable based on loss of refractility. Quantitative analysis showed that diapedesis of both control and ERM-deficient T cells was infrequent on untreated monolayers (Figure 6B). In contrast, approximately 50% of T cells applied to TNF-α treated monolayers passed through during the 2 hour imaging period. No difference in rate or frequency of diapedesis was observed between wild-type and ERM-deficient T cells.

In the high endothelial venules, T cells must decrease in velocity, adhere to the endothelium and extravasate under shear stress [46]. To assess diapedesis of ERM-deficient T cells under more physiologic conditions [25], we adapted the previous assay to include shear stress using a flow chamber [47]. The addition of shear stress dramatically reduced the frequency of T cell diapedesis. However, wild-type and ERM-deficient T cells crossed the monolayer at comparable frequencies (Figure 6C and Video S3). Thus, even under shear stress conditions, ERM proteins were not required for passage between endothelial cells *in vitro*.

## Discussion

Using mature T cells lacking both ezrin and moesin, we show for the first time that ERM protein expression is required for efficient T cell adhesion and trafficking. *In vivo*, we find that ERM deficient T cells exhibit striking defects in homing to lymphoid organs, but those cells that reach the lymph node show normal motility. Consistent with this, our *in vitro* studies show that ERM-deficient T cells can chemotax, cross endothelial barriers, and migrate efficiently in 3D collagen gels. Interestingly, we find that loss of ERM protein expression is associated with defects in β1, but not β2 integrin dependent responses. Our results show that ERM proteins promote T cell trafficking, and shed new light on the relevant steps where ERM protein expression is required.

Using experimental approaches ranging from transwell assays to 2-photon imaging of lymph nodes, we find that ERM proteins are largely dispensable for T cell locomotion and chemotaxis. Indeed, deleterious effects of ERM protein deletion were only detectable in transwell assays with a 3 µm pore size membrane. These findings contrast with several studies showing that expression of constitutively active and hyperphosphorylated forms of ERM proteins impair T cell migration [30], [39], [41]. This difference likely reflects dominant negative effects of constitutively active ERM proteins, and/or a requirement for ERM protein cycling. T cells expressing excess active ERM proteins exhibit enhanced cortical stiffness and diminished cell deformability, which likely impedes migration in ways that do not occur in T cells lacking ERM proteins. Indeed, we observed that ERM-deficient T cells actually migrated somewhat better than control cells in some experimental settings. Enhanced migration was also observed in LOK-deficient T cells, where ERM proteins are hypophosphorylated [39]. Interestingly, we find that the effects of ERM protein loss are context dependent. In transwell assays, loss of ERM proteins did not enhance migration, and actually impaired migration through small pores. On the other hand, ERM-deficient T cells migrated normally within the lymph node stroma, and in collagen gels, they showed slightly enhanced velocity and increased chemotactic directionality. This might reflect a difference in the physical challenge the cells confront; localized cortical rigidity might be important in a transwell setting where the cell must push against itself, but enhanced deformability may be advantageous when there is a 3D matrix to push against, and a complex path to navigate. In addition, migration through small transwell pores may depend in part on integrin-based adhesion to serum fibronectin deposited on the membrane, whereas migration within confined 3D spaces can occur in the complete absence of integrins [48]. Regardless of the mechanistic basis for this difference, our data support a model in which ERM proteins promote migration in some, but not all situations. Taken together with the literature on the effects of altering ERM protein phosphorylation, our data further indicate that unlocking the cortical cytoskeleton by transient ERM protein de-activation is essential for adhesion and migration.

In our system, ezrin protein is undetectable and moesin protein is reduced by 80–90% ([37] and data not shown). Nonetheless, a small amount of moesin remains and under some circumstances, we have observed compensatory hyperphosphorylation [37]. Thus, it remains possible that the remaining residual moesin is sufficient to support T cell migration. This caveat is particularly relevant for the *in vivo* imaging studies where analysis was limited to the small population of cells that were able to reach the lymph node. Since the remaining moesin was insufficient to support several T cell functions, we favor the interpretation that cell migration does not require ERM protein expression. However, definitive resolution of this question will ultimately require the generation of mice genetically deficient for both ezrin and moesin.

Liu et al showed that T cells expressing moderate levels of constitutively active ERM proteins exhibit enhanced integrin-dependent adhesion [30]. In keeping with this, one of the most striking phenotypes we observed in ERM-deficient T cells was diminished adhesion and uropod formation in response to β1 integrin ligands. Although adhesion defects were not observed in LOK^−/−^ T cells [39], these cells retain ∼50% residual phospho-ERM protein expression, which may be sufficient to support integrin function. Interestingly, the adhesive defects we observed in ERM-deficient T cells were limited to β1 integrins; adhesion and uropod formation in response β2 integrins engagement was normal. Normal β2 integrin function is also observed in the TCR signaling pathway, since we showed previously that ERM-deficient T cells form a normal immunological synapse with APCs [37]. The β1 integrin-specific phenotype we observed differs from the phenotype of T cells expressing constitutively active ezrin, in which adhesion via both β1 and β2-integrins is enhanced [30]. In that system, the constitutively active proteins may induce high basal association of integrins with the cortical cytoskeleton, thereby affecting the adhesive response at a global level.

Additional study will be required to identify the mechanisms through which ERM proteins promote β1 integrin function. Although the pathways leading to integrin activation are still largely ill-defined, most of the known intermediates are required for the activation of both β1 and β2 integrins [49], [50], [51], [52], [53]. Nonetheless, the existence of distinct β1 and β2 integrin signaling pathways is supported by several studies [54], [55], [56]. One key intermediate that is required for activation of both β1 and β2 integrins is the small GTPase Rap1 [57], [58], [59], [60], [61], [62], [63]. However, we tested chemokine-induced Rap-1 activation in ERM-deficient T cells and observed no defects (data not shown). Moreover, involvement of Rap1 cannot explain the β1 integrin-specific effect we observe. Indeed, one study suggests that Rap1 is required for β2, but not β1 integrin activation [55]. The best evidence for β1-specific intermediates comes from the work of Mayadas and coworkers, who showed that PKC signaling was only required for β1-dependent adhesion in T cells responding to SDF-1α or PMA [55]. A link between ezrin and PKC signaling has also been demonstrated during wound healing in fibroblasts, where ERM phosphorylation is dependent on the catalytic function of PKC, and ezrin co-sediments with both PKC and β1 integrins [64]. Thus, the defects we observe in ERM-deficient cells could lie downstream of PKC signaling. In support of this model, we observe clear defects in the ability of PMA to stimulate β1-dependent adhesion in ERM-deficient cells.

Given the defects in β1-integrin responses, we were surprised to find that ERM-deficient T cells could efficiently cross an endothelial monolayer expressing VCAM-1 in our in vitro diapedesis assays. This is probably not due to compensation by β2-integrins, since the endothelial cells used for these assays do not express ICAM-1 or other known β2 integrin ligands. Nonetheless, the possibility remains that other cell adhesion molecules can promote diapesesis in our assay. In vivo, β1-integrin ligands are involved in T cell migration to peripheral tissues, particularly inflammatory environments [65]. It will be interesting to test whether loss of ERM protein expression affects T cell migration in an in vivo inflammatory response.

While we have not tested T cell migration in an inflammatory setting, we did find defects in homing of ERM-deficient T cells to lymphoid organs, a process that is β2-integrin dependent [66], [67]. Our data show that the decrease in ERM-deficient T cell traffic to lymphoid organs under homeostatic conditions is offset by accumulation in the blood, indicating that at least one component of the defect is a failure to exit the circulation. Although we did not observe diminished β2-integrin responses *in vitro*, the possibility remains that ERM proteins are needed to cross the HEV *in vivo*. Another possibility is that ERM-deficient T cells may have an abnormally short dwell time in lymphoid organs. Indeed, Liu et al. demonstrated that T cells expressing constitutively active ERM proteins exhibit diminished diapedesis across the HEV *in vivo* as well as diminished rates of exit from lymphoid organs [30]. Although the kinetics of T cell ingress and egress from lymphoid organs should be measured directly, we note that our assays were carried out only 1 hour after transfer, when the effect of altered exit rates is expected to be minimal. By the same argument, we do not expect that differential cell survival is a major contributor to the phenotype we observe. In the complex context of *in vivo* responses, it is worth noting that ERM proteins participate in the function of other receptors such as CD43 and CD44 that promote T cell adhesion and migration. In particular, Li et al [40] have shown that hyperphosphorylation of ERM proteins, either in cells expressing constitutively active mutants or in T cells from Lupus patients, result in enhanced adhesion through the hyaluronic acid receptor CD44, a known ERM binding partner. Moreover, interactions of PKC and ERM proteins with both CD43 and CD44 have been shown to promote T cell migration [68], [69]. These functions may contribute to the defects in T cell homing we observe *in vivo*. Finally, it is important to point out that our system for generating ERM-deficient T cells relies on the use of previously activated cells, which do not re-circulate in the same way as naïve T cells. Additional, more definitive analysis of T cell trafficking should be carried out using naïve T cells bearing genetic deletion of both ezrin and moesin.

Increased ERM protein phosphorylation has been observed in Lupus patients [40], and enhanced ERM protein expression and phosphorylation is associated with leukemia and lymphoma [70], [71]. Conversely, stress hormones induce loss of ERM proteins, leading to reduced T cell migration and activation [72]. Our results showing that ERM proteins are required for efficient adhesion and trafficking of T cells help to explain why changes in ERM proteins are associated with immunopathology, and will facilitate the use of these proteins as therapeutic targets.

## Materials and Methods

### Ethics Statement

All studies involving animals were carried out according to guidelines put forth by the NIH Guide for the Care and Use of Laboratory Animals, as approved under protocol #2008-10-667 by the Children's Hospital of Philadelphia Institutional Animal Care and Use Committee.

### Mice

C57Bl/6 mice were obtained from Jackson Labs. Homozygous ezrin^lox/lox^ mice [73] provided by Dr. Andrea McClatchey (Massachusetts General Hospital) were crossed with CD4-cre transgenic mice on the C57Bl/6 background (Taconic Farms, Inc.) to produce ezrin^flox/flox:CD4cre^ mice with conditional deletion of ezrin in mature T cells, as described previously [37]. Littermates or C57Bl/6 mice were used as wild-type controls.

### Cell culture

All tissue culture reagents were from Invitrogen. Murine peripheral lymph node CD4+ T cells were isolated by negative selection using anti-CD8 (2.43) and anti-MHCII (M5/114.15.2). T cells were stimulated in 24-well plates coated with 1 ug/mL anti-CD28 (PV1) and anti-CD3 (2C11) (both from BioXCell) for three days and maintained in DMEM supplemented with 5% FBS, penicillin, streptomycin, glutaMAX, Hepes, NEAA and β-mercaptoethanol at 37°C, 10% CO_2_. T cell blasts were then rested for two days before siRNA suppression, or up to 5 days for experimental use. 3B-11 murine vascular endothelial cells (ATCC) were maintained in DMEM supplemented with 10% FBS, penicillin, streptomycin and glutaMAX for up to 25 passages.

### siRNA-mediated protein suppression

SiRNA duplexes against moesin were purchased from Dharmacon RNAi Technologies. The two targeting sequences used were GGAGCGUGCUCUCCUGGAAUU (siMoesin 1) and CGGUCCUGUUGGCUUCUUAUU (siMoesin 2) [37]. SiControl #2 was used as a control. To produce ezrin and/or moesin deficient murine CD4+ T cell blasts, freshly isolated ezrin^flox/flox^ or ezrin^flox/flox:CD4cre^ T cells were stimulated as described above and removed from stimulation for two days. Cells were then washed and resuspended in unsupplemented DMEM. Cells (10^7^) were mixed with 500 pmol of either siMoesin or siControl in 4 mm gap cuvettes and transfected using a BTX ECM830 electroporator at 290 V for 10 ms. After transfection, cells were maintained in supplemented DMEM and 100 U/mL rhIL-2 (obtained through the AIDS Research and Reference Reagent Program, Division of AIDS, NIAID, NIH: rhIL-2 from Dr. Maurice Gately, Hoffmann-La Roche Inc.) for 48–72 hours.

### Flow cytometry

Single-cell suspensions were stained with the following fluorescently conjugated mAbs: CCR7 (CD197)-APC, CD18-PE, and CD29-PE (Biolegend). Flow cytometry and analysis was done using FlowJo (Tree Star). Dead cells were excluded based on forward scatter and side scatter analysis.

### Transwell assays

CD4+ ezrin and/or moesin deficient or control T cell blasts were washed and resuspended in 1% FBS DMEM and rested for 1 hour at 37°C, 10% CO_2_. Lower chambers of 96-well, 3 or 5 µm pore ChemoTx plates (Neuro Probe) were filled with 1% FBS DMEM or 1% FBS DMEM with 40 nM CCL19 (R&D Systems). After resting, cells (5×10^5^) were added to the upper chambers and the plates were incubated in a humidified chamber at 37°C, 10% CO_2_ for 2 hours. Membranes were washed and the plate centrifuged to collect cells in the lower chamber. Cells were counted using a hemocytometer, and percent of input cells reaching the lower chamber was calculated.

### Analysis of chemotaxis in 3D collagen gels

Analysis of chemotaxis in 3D collagen gels was performed using Ibidi μ-slide chemotaxis 3D chambers. To produce 3D collagen gels for migration studies, bovine skin collagen Type I (Purecol, Advanced Biomatrix) was used at either 1.6 mg/ml for 5 µm pores, or 2.0 mg/ml for 2 µm pores [74]. The collagen solution was neutralized and mixed with T cell blasts according to the manufacturer's instructions, to yield a final concentration of 1× DMEM, 1.2% FBS, 0.3% NaHCO_3_ and 1.6×10^6^ T cells/ml. The collagen-cell suspension was loaded into tissue culture treated μ-slide chemotaxis 3D chambers and allowed to polymerize at 37°C, 5% CO_2_ for 1 hour. 80 nM CCL19 in DMEM containing 1% FBS was added to one reservoir of each chamber and DMEM containing only 1% FBS was added to the opposite reservoir. Chambers were incubated for 30 minutes at 37°C to allow establishment of a linear chemokine gradient. Multiple chemotaxis chambers were visualized in parallel using a 5× objective on a Zeiss Axiovert 200 M inverted microscope equipped with an MS-2000 automatic stage (Applied Scientific Instruments) and a 37°C environmental chamber. Images were collected at 1-minute intervals using Slidebook 5.0 (Intelligent Imaging Innovation), and exported to Volocity 6.0 (Perkin-Elmer) for analysis. Cells were detected using the 2D objects tool, and tracked automatically. Analysis was restricted to motile T cells by eliminating objects that were present in the field of view for less than 30 minutes and those that displaced less than 150 µm. Population analysis of unfiltered data did not reveal significant differences in the proportion of motile T cells in control and experimental samples.

### In vivo homing assays

CD4+ ezrin and moesin deficient or control T cell blasts were washed and resuspended at 2×10^7^ cells/mL in PBS. Cell suspensions were combined with an equal volume of either 1 µM CFSE or 5 µM CellTracker Orange CMTMR (both from Invitrogen) and incubated at room temperature for 5 minutes (for CFSE) or at 37°C for 15 minutes (for CMTMR). Cells were then resuspended at 1×10^8^ cells/ml in PBS, combined, and 200 µl (2×10^7^ cells) were injected intravenously into the tail veins of C57Bl/6 hosts. After 1 hour, recipient mice were sacrificed, and blood, spleen, peripheral and mesenteric lymph nodes were harvested. Cells were stained for surface markers and assessed by flow cytometry.

### Intravital microscopy

Cells were prepared and transferred to recipient mice as described for *in vivo* homing assays. After 1 hour, recipient mice were sacrificed and peripheral lymph nodes were harvested, placed in a heated chamber, and perfused with warm oxygenated media (phenol-red free RPMI 1640 supplemented with 1% FBS). Cells were visualized using a 20× water-dipping lens on a Leica SP5 multi-photon microscope (Leica Microsystems) equipped with a Chameleon femtosecond pulse laser (Coherent) tuned to 920 nm. Z stacks with ∼5 µm step size and 50 µm total thickness were collected every minute for 40 minutes. Migration analysis was performed using object detection and tracking functions in Volocity 6.0. Migrating cells were defined as cells that displaced at least two cell lengths or exhibited a minimum average velocity of 2 µm/minute.

### Adhesion Assays

Adhesion assays were performed in triplicate as previously described [53]. 96-well COSTAR plates were coated with fibronectin (R&D Systems) at 0.3, 1.0, 3.0 or 10 µg/ml or 96-well NUNC MaxiSorp plates were coated with recombinant mouse ICAM-1/Fc chimera (R&D Systems) at 0.5, 1.5 or 5 µg/ml overnight at 4°C. Wells were blocked with 2.5% BSA in PBS with Ca^++^ and Mg^++^ for 1 hour at room temperature before use. All cells and reagents were suspended in 2.5% BSA in PBS with Ca^++^ and Mg^++^. Cells were stained with 2.5 µg/ml calcein-AM (Invitrogen) for 30 minutes at 37°C, washed and resuspended in 2.5% BSA in PBS with Ca^++^ and Mg^++^. Cells were then incubated on ice with or without 10 µg/ml anti-CD3 biotin (BioXCell). Cells (1×10^5^) were then added to each well and allowed to settle on ice for 1 hour. Plates were read on a fluorescent plate reader at 485 nm excitation and 528 nm emission filters for total florescence. To initiate TCR crosslinking, streptavidin was added to each well. Alternatively, to bypass TCR signaling, PMA was added to a final concentration of 5 ng/ml. Cells were warmed to 37°C for 10 minutes, washed 3 times with cold blocking buffer and read again.

### Uropod Assessment

12 mm round glass coverslips were acid washed with 10% H_2_O_2_ in 0.1 N HCL, rinsed in ddH_2_O followed by methanol, and flame dried. Coverslips were coated with either 50 µg/ml of human fibronectin (R&D Systems) for 2 hours at room temperature, or 50 µg/ml anti-hIgG Fc (ICN Biomedicals) overnight at 4°C before being additionally coated with either 3 µg/mL rVCAM-1 or 1 µg/ml rICAM-1 Fc (R&D Systems) for 2 hours at room temperature, and washed with PBS before use. T cells (1.5×10^5^) were resuspended in 50 µl unsupplemented DMEM, added to each coverslip and allowed to settle in a humidified chamber for 30 minutes at 37°C. Unbound cells were washed off and bound T cells were fixed using 3% paraformaldehyde/PBS. Cells were imaged using a Zeiss Axiovert 200 M microscope with a 63× objective and DIC optics. To quantify uropod formation, at least 50 randomly selected cells per coverslip were scored based on an elongated shape and the presence of a distinct tail-like structure.

### Diapedesis assays

To assay diapedesis in the absence of shear flow, 3.5×10^5^ 3B-11 endothelial cells were seeded in 35 mm tissue culture dishes (Corning) and cultured overnight to form a confluent monolayer. Media was replaced the following morning with DMEM containing 0.25% FBS. After 8 hours of serum deprivation, cells were either left unstimulated, or stimulated with 50 ng/mL TNF-α (PeproTech) for an additional 16 hours. Ezrin and moesin-deficient or control CD4+ T cell blasts were resuspended in DMEM containing 0.25% FBS, 25 mM HEPES, pH 7.0, and 1.4×10^6^ T cells were added to each dish. Cells were imaged in a humidified 37°C environmental chamber mounted on a Zeiss Axiovert 200 M microscope, using a 20× long working distance objective and DIC optics. Images were collected every 30 seconds for 2 hours using Slidebook 5.0. Using these optics, T cells undergoing diapedesis lose their rounded, refractile morphology and become flattened and dark. Alternatively, control and experimental T cells were analyzed in parallel using fluorescence microscopy. For this, ezrin and moesin deficient or control CD4+ T cell blasts (2×10^7^/mL) were washed, resuspended in PBS, and labeled with either 2 µM CFSE or 10 µM CMTMR as described above (controls were performed with reciprocal staining). Cells were mixed in a 1∶1 ratio and added to the 3B-11 monolayers, which were grown on μ-Dish 35 mm, high dishes (Ibidi). Cells were imaged at 20× using DIC optics to visualize the endothelium and assess diapedesis, and fluorescence optics to distinguish the two cell populations. In both assay variants, quantitation was performed by monitoring all cells that were present in the initial few minutes of each sequence, and scoring the percent that underwent diapedesis within 2 hours.

Analysis of diapedesis under shear flow conditions was carried out using the BioFlux System (Fluxion Biosciences). 48-well low shear Bioflux plates were coated with 50 µg/ml of fibronectin (R&D systems) for 30 minutes at 37°C, washed with PBS and seeded with 3B-11 cells (1×10^7^/mL) in supplemented DMEM for 2 hours at 37°C, 10% CO_2_. 3B-11 cells were then grown overnight in supplemented DMEM under passive flow. Supplemented DMEM was then replaced with DMEM containing 0.25% FBS. After 6 hours of serum deprivation, cells were either left unstimulated, or stimulated with 50 ng/mL TNF-α (PeproTech) for an additional 6 hours. Ezrin and moesin deficient or control CD4+ T cell blasts were labeled with either 2 µM CFSE or 10 µM CMTMR (as described above), mixed in a 1∶1 ratio, and resuspended in DMEM supplemented with 0.25% FBS, 25 mM HEPES, pH 7.0. T cells (5×10^6^/mL) were perfused over the monolayer at a shear stress of 0.24 dyne/cm^2^ for approximately 2 minutes to allow cell adhesion to the endothelium. The wall shear-stress was then increased to 0.5 dyne/cm^2^ and images were taken every 30 seconds for 1 hour on a 37°C heated plate using a 10× objective on an ECLIPSE TE2000-U microscope (Nikon) with brightfield optics. Quantitation was performed by monitoring all cells that were present in the frame for at least 20 minutes, and scoring the percent that underwent diapedesis.

## Supporting Information

Video S1 Intravital imaging of ERM-deficient T cells in lymph node explants. CMTMR stained wild-type and CFSE stained ERM-deficient T cells were mixed in a 1∶1 ratio and injected into the tail veins of C57Bl/6 hosts. Lymph nodes were harvested 1 hour after injection, and imaged by multi-photon microscopy. A 2D projection of the 3D dataset is shown.
(MOV)

Video S2 Diapedesis under static conditions. Confluent monolayers of 3B-11 endothelial cells were treated with TNF-α to upregulate VCAM-1. Wild-type T cells were added to the apical surfaces. Cells were imaged at 37°C using DIC optics, with images collected every 30 seconds for 2 hours.
(MOV)

Video S3 Diapedesis in the presence of shear stress. Confluent monolayers of 3B-11 endothelial cells grown under passive flow were treated with TNF-α to upregulate VCAM-1. CMTMR stained wild-type T cells (red) and CFSE stained ERM-deficient T cells (green) were mixed in a 1∶1 ratio and added to the apical surfaces. Cells were imaged at 37°C, with sequential brightfield and fluorescence images collected every 30 seconds for 1 hour. Images were superimposed for analysis. White arrows indicate wild-type and ERM-deficient T cells on the apical surface of endothelial cells. Arrows change to red when the indicated cells pass through the endothelial monolayer and continue migrating along the basal surface.
(MOV)
